# Increased intestinal permeability and gut dysbiosis in the R6/2 mouse model of Huntington’s disease

**DOI:** 10.1038/s41598-020-75229-9

**Published:** 2020-10-26

**Authors:** Tiberiu Loredan Stan, Rana Soylu-Kucharz, Stephen Burleigh, Olena Prykhodko, Ling Cao, Naomi Franke, Marie Sjögren, Caroline Haikal, Frida Hållenius, Maria Björkqvist

**Affiliations:** 1 Wallenberg Neuroscience Center, Brain Disease Biomarker Unit, Department of Experimental Medical Sciences, BMCA10, Lund University, 22184 Lund, Sweden; 2 Department of Food Technology, Engineering and Nutrition, Lund University, Lund, Sweden; 3 Neural Plasticity and Repair UnitDepartment of Experimental Medical Science, BMC A10, Wallenberg Neuroscience Center, 221 84 Lund, Sweden

**Keywords:** Diseases, Neuroscience, Diseases of the nervous system, Huntington's disease, Microbiota

## Abstract

Huntington’s disease (HD) is a progressive, multifaceted neurodegenerative disease associated with weight loss and gut problems. Under healthy conditions, tight junction (TJ) proteins maintain the intestinal barrier integrity preventing bacterial translocation from the intestinal lumen to the systemic circulation. Reduction of TJs expression in Parkinson’s disease patients has been linked with increased intestinal permeability—leaky gut syndrome. The intestine contains microbiota, most dominant phyla being *Bacteroidetes* and *Firmicutes*; in pathogenic or disease conditions the balance between these bacteria might be disrupted. The present study investigated whether there is evidence for an increased intestinal permeability and dysbiosis in the R6/2 mouse model of HD. Our data demonstrate that decreased body weight and body length in R6/2 mice is accompanied by a significant decrease in colon length and increased gut permeability compared to wild type littermates, without any significant changes in the protein levels of the tight junction proteins (occludin, zonula occludens). Moreover, we found an altered gut microbiota in R6/2 mice with increased relative abundance of *Bacteroidetes* and decreased of *Firmicutes*. Our results indicate an increased intestinal permeability and dysbiosis in R6/2 mice and further studies investigating the clinical relevance of these findings are warranted.

## Introduction

Huntington’s disease (HD) is an autosomal hereditary neurodegenerative disease caused by an elongation of CAG repeats of variable length in the gene encoding the protein huntingtin (HTT)^1^. In addition to motor dysfunction^2^, HD patients exhibit symptoms including cognitive decline^3^, depression, anxiety, irritability as well signs of inflammatory bowel disease^4^.

The gastrointestinal system houses millions of microorganisms, collectively called the gut microbiome. Under healthy conditions, the gut serves as a barrier preventing bacterial translocation from the intestinal lumen into the systemic circulation. The mucus layer is not impenetrable and a second and crucial layer of protection is maintained by tight junctions (TJs) between epithelial cells, which consist of transmembrane, scaffold and adaptor proteins^5^. TJ proteins include occludin, claudin and zonula ocludens (ZO)^6^. Similarly to HD, in Parkinson’s disease (PD), gastrointestinal (GI) impairment is a frequently reported early symptom of the disease and its involvement in disease progression is a growing topic of research^7^. Clinical research studies have indicated a significantly lower expression of occludin, but not ZO-1, in colonic samples from PD patients as compared to controls^8^. Moreover, the cellular distribution of both occludin and ZO-1 has been found significantly altered in colonic mucosal samples from PD patients^8^. Previous studies performed in the R6/2 HD mouse model have indicated abnormalities at gut level: gut motility is altered, with increased transit time for the food to travel through the gut^9^. In a more recent study, it was shown that impairment of blood–brain barrier is an early event in R6/2 mice and is due to alterations in TJs expression, in particular expression of occludin^10,11^. Also, it has been shown that R6/1 male mice have gut dysbiosis, namely an increased relative abundance of *Bacteroidetes* (Gram −) and lower relative abundance of *Firmicutes* (Gram +)^12^. It has been indicated that gut dysbiosis could play a role in modulating physiological, behavioural and motor abnormalities in neurological diseases^13–15^.

In this study we aimed to investigate whether the integrity of the GI tract in the R6/2 mouse model of HD is changed, whether this is linked to GI tight junction expression and if gut microbiota composition is altered.

## Methods

### Animals

All the experimental procedures were performed in agreement with the approved guidelines in the ethical permits approved by the local Animal Ethics Committee Malmö/Lund Animal Welfare and Ethics Committee (ethical permit numbers M72/16 and 2505/18). Experiments were carried out on 12, 16 and 18 weeks old male transgenic R6/2 HD mice (expressing exon 1 of the HD gene)^16^ and their wild type (WT) littermates. 12 weeks correspond to early stage disease and 16/18 weeks to an early/mid stage disease in our colony of mice^17^. Mice were obtained through crossing heterozygous R6/2 males with WT females (F1 of CBAxC57BL/6J). Genotyping was performed as described previously^16^. In order to determine the CAG-repeat length, tail tips were sent on dry ice to Laragen (Laragen Inc., CA, USA). The CAG-repeat lengths of the R6/2 mice used in this study ranged between 242 and 257, resulting in a disease progression slower than that of the R6/2 mouse with 150 CAG repeats as described previously^18^. Unless stated otherwise, mice were group-housed (2–5 animals) in universal Innocage mouse cages (InnoVive, San Diego, CA, USA) enriched with nesting material and kept under 12 h light/dark cycle, 22 °C, with ad libitum access to water and normal chow diet.

### Body weight, body length, serum glucose levels and intestine length assessments

Prior to tissue collection/intestinal permeability assessment, the animals were weighed. Next, the animals were anesthetized with pentobarbital intraperitoneal injection (Apoteket, Lund, Sweden). Animals were placed thereafter in a supine position and the body length was assessed with a ruler by measuring the distance from the nostrils to the anus. Blood was collected from the right heart ventricle using a wide-bore needle (18G/40 mm) to prevent applying suction force. To determine the glucose serum levels, one small drop of blood was placed on a single-use Bayer Contour Next (Apoteket, Lund, Sweden) test probe and the value was immediately read on the Bayer Contour Next (Apoteket, Lund, Sweden) display. The mice were euthanized, and intestine length was assessed after opening the peritoneum. In brief, the GI tract was untangled, gently flushed with 0.9% saline solution, and the small intestine and colon measured separately.

### Intestinal permeability

For analysis of intestinal permeability, we performed experiments similarly as to previously reported^19^. In brief, animals were water-deprived for approximately twelve hours and the body weight was assessed in the morning of the following day. Fluorescein isothiocyanate–dextran (FD4), average molecular weight 3000–5000 (Sigma-Aldrich), was dissolved in 1% phosphate buffered saline (PBS) and administered to each mouse (44 mg/100 g body weight) delivered at a volume of 10 µl/1 g body weight with a mouse gavage needle attached to a 1 ml syringe. Mice were sacrificed after 4 h and blood samples collected. Blood samples were stored at room temperature for 30 min, protected by light, and then centrifuged at ~ 2500×*g* for 15 min to obtain serum. Serum was collected and kept dark at 4 °C for up to six hours. Thereafter, serum was diluted with an equal volume of PBS. 100 μl of diluted serum was added to a 96-well microplate in duplicates. The concentration of FD4 in serum was determined by spectrophotofluorometry with an excitation of 492 nm using as standard serially diluted FD4 (0, 125, 250, 500, 1000, 2000, 4000, 6000, 8000 ng/ml).

### Western blot

Animals were anesthetized by an overdose of sodium pentobarbital (Apoteksbolaget, Lund, Sweden), and the brain (cortex) and colon were dissected and frozen in dry ice. The cortex samples were lysed in lysis buffer (Tris pH 8, 10 nM, EDTA 1 mM, 1% SDS (sodium dodecyl sulfate), glycerol 12.5%) containing anti-protease and anti-phosphatase solutions (ThermoFisher Scientific) and further followed the blotting protocol as previously described^20^. The proximal colon tissue samples were homogenized in Radioimmunoprecipitation assay buffer (RIPA buffer, Abcam, ab156034) containing 1% SDS and protease inhibitor cocktail (1 tablet per 7 ml, EDTA-free cOmplete Roche, Sigma-Aldrich) using a sonicator (at %50 amplitude). Samples were centrifuged at 14,000×*g* for 10 min at 4 °C. The supernatant was collected, and protein concentration was measured (DC Protein Assay, BioRad). 20 μg proteins were fractionated on a 4–14% SDS–polyacrylamide gel electrophoresis gel and transferred to either 0.2 μm nitrocellulose (Bio-Rad, #1704158) or polyvinylidene difluoride (PVDF) membranes (Bio-Rad, #1704156). Membranes were blocked in TBS-0.1% Tween-20 (TBS-T) with 5% non-fat dry milk for 2 h at room temperature, then probed with antibodies to occludin (anti-rabbit, ab216327, 1:1.000), or ZO-1 (anti-rabbit, Invitrogen #61–7300, 1:500), β-actin (anti-mouse, Sigma-Aldrich A3853, 1:10.000) overnight at 4 °C. After washing the membranes three times with TBS-T, membranes were incubated for 2 h at room temperature with a goat anti-rabbit horseradish peroxidase (HRP) conjugated secondary antibody (Dako, # 1:10.000), or anti-mouse HRP conjugated secondary antibody (Dako, # 1:10.000). After extensive washing, the membranes were developed using Clarity Western ECL Substrate (Bio-Rad, #1705060) according to the manufacturer’s instructions. The membranes were imaged using the ChemiDoc MP Imaging System (Bio-Rad), and the data was exported as a 16-bit tiff file. The images were analysed with ImageJ version v.1.52A (NIH, USA) using the Gel Analyser tool.

### Immunohistochemistry and histopathology

Mice were perfused as previously reported^19^. The medial colon segments were paraffin-embedded and cross-sectioned at 6 µm thickness. The sections were further processed in order to visualise tissue morphology and occludin localisation according to a hematoxylin and eosin (H&E) staining protocol^21^, in combination with occludin staining (anti-rabbit, Abcam, ab216327, 1:200). In brief, the paraffin-embedded sections were dried for 60 min at 60 °C and deparaffinized in a series of xylene and ethanol baths. Thereafter, sections were treated in 80% formic acid for 10 min and washed with dH_2_O. Antigen retrieval was performed at 95 °C in 0.01 M citrate buffer pH 8.5, whereas the endogenous peroxidase was blocked with 3% H_2_O_2_ for 15 min. After the wash steps, the sections were blocked in 5% normal goat serum (NGS), 2% (bovine serum albumin) BSA (VWR, Sweden) and 0.25% Triton-X in PBS and probed overnight at 4 °C with the primary antibody occludin (anti-rabbit, ab216327, 1:200). The following day sections were washed, incubated for 2 h with the secondary antibody (1:6000 Dnk anti-Rb IgG HPR Abcam, ab16284) at room temperature. Thereafter, samples were rinsed in PBS 3 times for 5 min before incubation for 1 h at room temperature with the amplification solution ABC (Avidin–Biotin complex). Further rinsing with PBS, the sections were incubated for 5 min with 3-3-Diaminobenzidine (DAB). Following the PBS rinse 3 times for 5 min, sections were dehydrated and cleared in a series of ethanol and xylene baths. Finally, sections were cover slipped with DPX mounting media (DPX mounting media, ThermoFisher Scientific, UK).

### 16S rRNA gene sequencing of the faecal microbiota: DNA extraction, PCR amplification and sequencing

DNA from faecal samples was extracted using the QIAamp PowerFecal DNA Kit (Qiagen, Sweden). Measurement of DNA concentration was performed using Qubit 2.0 Fluorometer (Thermo Fisher Scientific). The V4 region of 16S rRNA gene was amplified using forward and reverse primers containing Illumina overhang adaptors and unique dual indexes. The sequences of the 16S amplicon primers were (Forward)-5′ TCGTCGGCAGCGTCAGATGTGTATAAGAGACAGGTGCCAGCMGCCGCGGTAA and (Reverse)-5′ GTCTCGTGGGCTCGGAGATGTGTATAAGAGACAGGGACTACHVGGGTWTCTAAT^22^. Paired-end sequencing with a read length of 2 × 250 bp was carried out on a Miseq Instrument (Illumina, San Diego, USA) using a Miseq reagent kit v2 (Illumina, San Diego, USA). As an internal control, 5% of PhiX was added to the amplicon pool. Illumina sequencing adaptors were trimmed off during the generation of FASTQ files and reads that did not match any barcodes were discarded.

### Sequence analysis

Sequence data were analyzed with the open-source bioinformatics pipeline Quantitative Insights Into Microbial Ecology (QIIME)^23^. Sequences were removed when lengths were < 200 nucleotides, > 290 nucleotides or when the quality score fell below 25 as determined using PRINSEQ software^24^. After filtering, a total of 2,200,447 reads were obtained from 19 samples with an average of 115,814 reads per sample (min: 58,432 and max: 192,924). The sequences were normalized by rarefaction (depth of 58,400) using Qiime and grouped into operational taxonomic units (OTUs) at a minimum of 97% similarity by using Qiime’s closed reference method based on the Greengenes database (v.13.8), filtered by the removal of singletons and low abundance OTUs (minimum count fraction set at 0.001).

### Statistical analyses

We used GraphPad Prism 7.04 (GraphPad Software Inc., San Diego, CA, USA) to analyse data on body weight, body length, intestinal length, serum glucose levels, intestinal permeability, correlation of the permeability with microbiota, and protein expression. Data were verified first for Gaussian distribution with the D’Agostino-Pearson omnibus normality test. Depending on normality of data, Student's unpaired *t*-test or Mann Whitney test was used to analyse the intestinal permeability and protein expression. Results are presented as means ± SEM. Bonferroni correction was applied to correct for multiple testing. Spearman’s correlation coefficient was used to measure the statistical relationship between the body weight, intestinal permeability, serum glucose levels and members of gut microbiota. Differences with a *p* < 0.05 were considered statistically significant. A Qiime-based permanova (using the pseudo-F statistical test and 999 permutations) was used to test differences between R6/2 and WT microbiomes. OTU alpha/beta diversity (Shannon index) of the microbial community were applied to the OTU table at an even depth of 58, 400 sequences per sample. A partial least squares (PLS-X) loading variable and score scatter plot (bi-plot) was carried out using SIMCA software (version 15, Umetrics, Umeå, Sweden). The loading variables were the treatments, biomarkers and microbiome data, while the scores were the samples of the two genotypes (R6/2 and WT mice). LEfSe (Linear discriminant analysis effect size) was used to find microbial biomarker signals in the two genotypes^25^.

## Results

### Increased intestinal permeability in R6/2 mice

**Figure 1:**
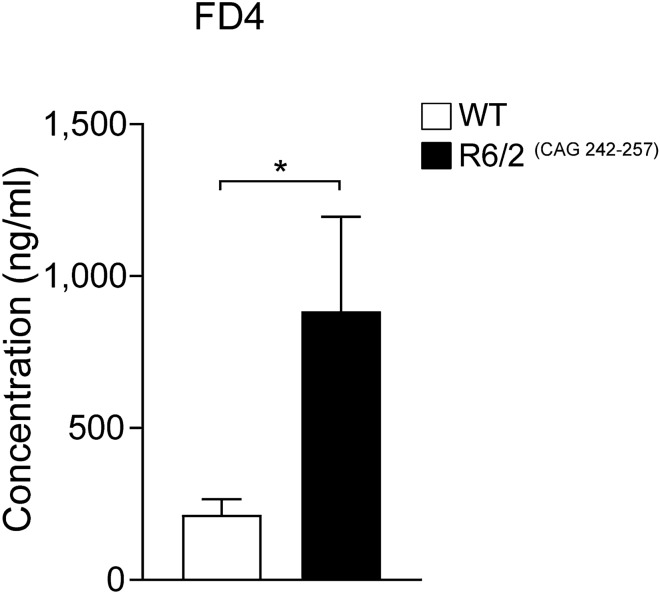
Intestinal permeability is increased in R6/2 mice. Intestinal permeability measured by determining the concentration of FD4 in the serum of R6/2 mice vs. wild type (WT) littermates at 16 weeks of age 4 h after oral FD4 administration. Data represent mean ± SEM, N = 10 for WT; N = 10 for R6/2; unpaired *t* test; *p ≤ 0.05. FD4 = Fluorescein Isothiocyanate-dextran.

In order to evaluate intestinal permeability, we utilized the R6/2 mouse model and assessed levels of FD4 in the circulation 4 h after oral gavage. R6/2 mice (16 weeks of age) display increased serum levels of FD4 compared to WT littermates, indicating that R6/2 mice have increased intestinal permeability (Fig. 1). In our study, we chose to evaluate R6/2 mice with a CAG repeat size of 242–257. This results in a slow progressing phenotype^17,18^ and at the time points chosen for this study, R6/2 mice are at an early (12 weeks) and an early-mid (16–18 weeks) disease stage. Similar to the previous findings^9^, we observed that R6/2 mice have a body weight reduction at week 12 of age and even more pronounced at 16 weeks. Moreover, in line with other studies^26^, we also found a decrease in R6/2 body length and increased blood glucose levels at 16 weeks of age (Table 1).

**Table 1:** Body weight, body length, intestinal length and serum glucose levels in R6/2 and wild type littermates at 12 and 16 weeks of age.

	WT		R6/2		P-value	
Age (weeks)	12	16	12	16	12	16
Body weight (g)	27.9 ± 0.9	30.0 ± 1.1	24.7 ± 0.6	19.5 ± 0.8	0.04	0.0012
Body length (cm)	8.9 ± 0.1	9.3 ± 0.01	9.0 ± 0.1	8.3 ± 0.0	0.5	0.002
Glucose levels (mM/L)	13.7 ± 1.0	15.02 ± 2.0	15.93 ± 1.7	26.6 ± 2.9	0.26	0.0013
Small intestine length (cm)	37.6 ± 1.0	35.1 ± 0.7	38.1 ± 1.0	36.8 ± 0.9	0.7	0.10
Colon length (cm)	8.7 ± 0.2	9 ± 0.2	7.6 ± 0.4	8 ± 0.2	0.06	0.01

R6/2 mice exhibit significantly reduced body weight and body length at 16 weeks of age. R6/2 mice have high blood glucose levels at 16 weeks of age. Colon length is significantly decreased at 16 weeks of age. N = 6–10 for WT; N = 6 for R6 /2; Mann–Whitney test; WT-wild type.

Interestingly, when looking at GI length, there were no significant changes of the small intestine length, regardless of age whereas the colon length was significant reduced at 16 weeks of age in the R6/2 vs. WT mice.

### Tight junction’s protein expression in R6/2 mice

**Figure 2:**
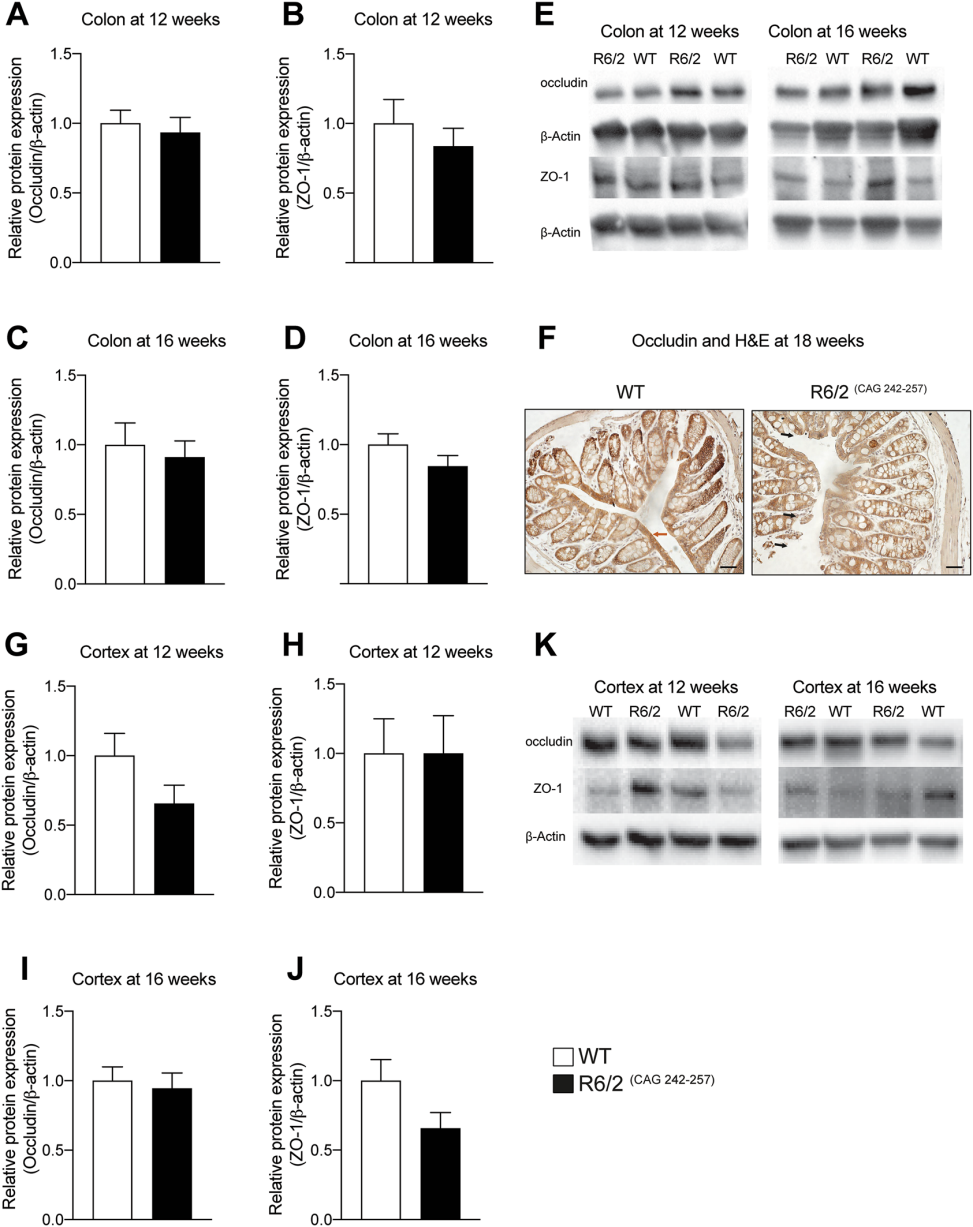
Tight junction protein expression is not altered in the colon and cortex of R6/2 mice. Quantitative western blot analysis showed no change in occludin (n = 5–7/genotype, p = 0.8763) **(A)** and ZO-1 (n = 5–7/genotype, p = 0.6389) **(B)** at 12 weeks and occludin (n = 6–7/genotype, p = 0.8357) **(C)** and ZO-1 (n = 5–8/genotype, p = 0.0653) **(D)** expression levels at 16 weeks in the proximal colon samples of R6/2 compared to WT mice. Western blot showing occludin and ZO-1 expression in the proximal colon samples of 12 and 16 weeks old R6/2 and WT mice **(E)**. Qualitative analysis of occludin expression in the colon of WT and R6/2 mice at 18 weeks of age. The occludin immunohistochemistry with H&E shows the reduced expression of occludin in R6/2 mice compared to WT mice (red arrows show an intact epithelium in WT colon and black arrows show disrupted epithelia in R6/2 colon) **(F)**. Assessment of occludin and ZO-1 protein expression levels showed no change in the cortex samples at 12 weeks **(G** and **H)** (occludin: n = 7/genotype, p = 0.1282, ZO-1: n = 7/genotype, p > 0.9999) and 16 weeks (occludin: n = 7/genotype, p = 0.62, ZO-1: n = 7/genotype, p0.0973) **(I**,**J)** in R6/2 compared with WT mice. **(K)** Western blot showing occludin and ZO-1 expression in the cortex of 12 and 16 weeks old R6/2 and WT mice. Data were normalized to β-actin protein expression and presented as a fold change in relation to WT animals. Non-parametric Mann–Whitney test was used for comparisons between the two groups. Data represent mean ± SEM. Scale bars represent 100 μm.

The reduction of the blood–brain barrier (BBB)’s TJ proteins was previously demonstrated in the R6/2 mice brain^10,11^. We therefore investigated the expression of TJ proteins, occludin and ZO-1, in both colon (Fig. 2A–E and Supplementary Fig. S1A-B) and cortex (Fig. 2G–K and Supplementary Fig. S1C-D) at 12 and 16 weeks of age using western blot. There was no difference in the expression of occludin and ZO-1 levels between R6/2 and WT mice neither in the cortex nor in the colon at 12 and 16 weeks. However, we observed reduced occludin expression in the colon mucosa of R6/2 mice and disrupted epithelial organization, whereas there was a strong expression of mucosal occludin and intact epithelium in WT mice at 18 weeks of age (Fig. 2F). Furthermore, H&E staining indicated an altered morphology and disrupted epithelial organization of colon mucosa in R6/2 mice whereas in WT the epithelium was intact at 18 weeks of age (Supplementary Fig. S2).

### Gut microbiota is altered in R6/2 mice

**Figure 3:**
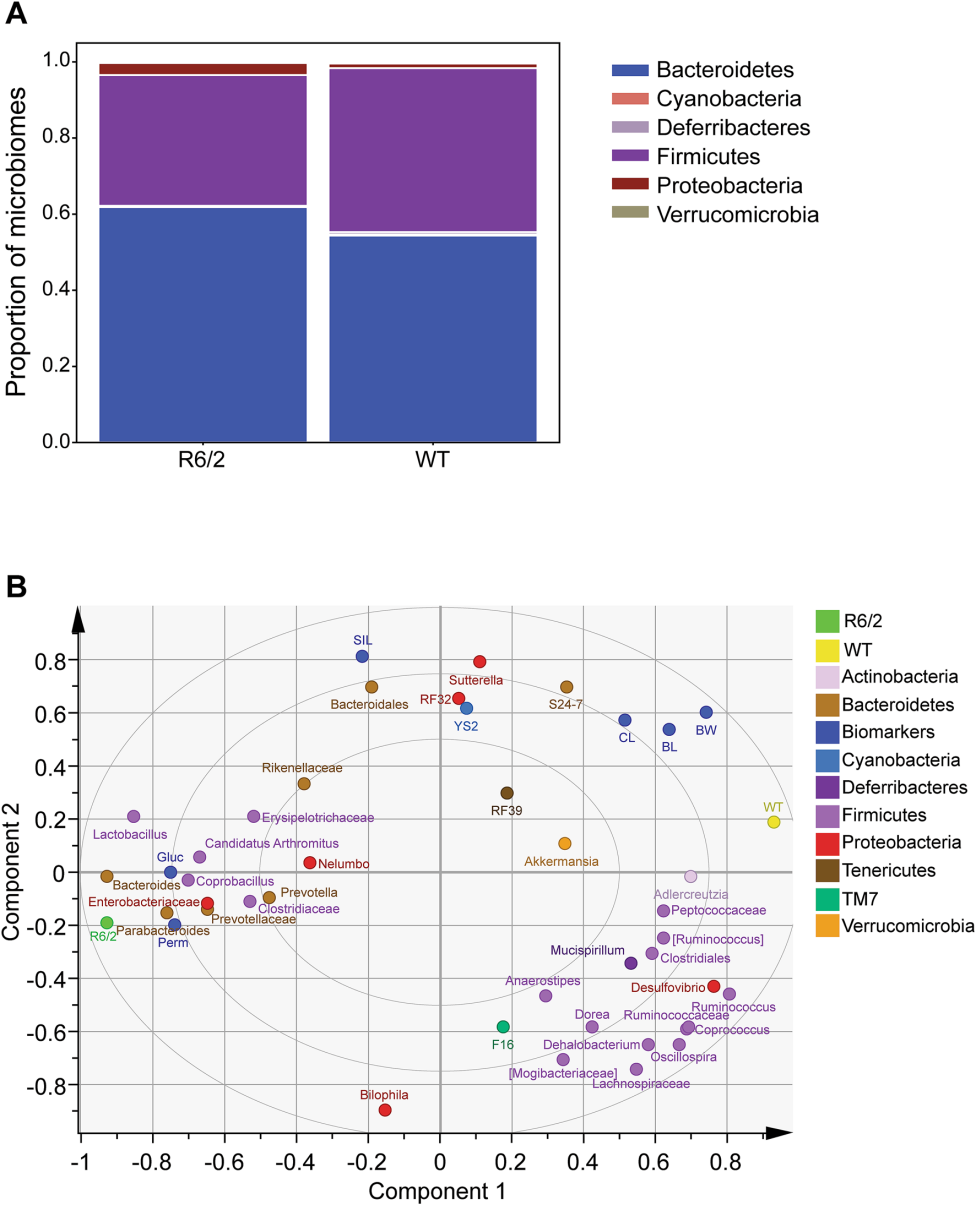
R6/2 and WT mice gut microbiota analysis. Gut microbiota composition at phylum and genus level using16S rRNA analysis of faecal samples from 16 weeks of age mice (n = 9–10/genotype) **(A)**. Partial least squares (PLS-X) loading and score scatter plots of different gut microbial taxa and biomarkers (blue) from R6/2 and WT mice (n = 4–6/genotype). *Firmicutes* taxa (purple) are associated with WT, while *Bacteroidetes* taxa (brown), FD4 permeability (Perm) and glucose levels (Gluc) are strongly associated with R6/2. R6/2 mice show an increased relative abundance of *Bacteroidetes* and a decreased relative abundance of *Firmicutes* (**B**).

It has been shown previously that R6/1 male mice display altered microbiota^12^. We therefore investigated if there is a difference in GI microbiota in our R6/2 mice at 16 weeks of age. We analysed bacterial diversity using 16S rRNA analysis of faecal samples from 16 weeks of age mice. The data showed that there were clear visual differences between R6/2 and WT taxonomic profiles (Fig. 3A), which were shown to be significant based on Qiime’s PERMANOVA analysis (*p* = 0.038). There was a higher relative abundance of *Bacteroidetes* (Gram −) and lower relative abundance of *Firmicutes* (Gram +) in R6/2 mice as compared to the WT (Supplementary Fig. S3A and relative abundance of OTUs assigned at the phylum level in Table S1). We performed Partial Least Squares (PLS) analysis in order to understand how different biomarkers and microbiota data correlate with either R6/2 or WT group (Fig. 3B). The correlation analysis indicated that permeability (Perm) and glucose (Gluc) were strongly associated with R6/2 group, while most of the other biomarkers (eg. colon length (CL), body length (BL) and body weight (BW)) were more associated with wild-type group. The gut microbiota composition differed significantly between groups, with clear associations of the R6/2 group with a number of bacteria, including *Bacteroides*, *Parabacteroides*, *Lactobacillus*, *Coprobacillus* and the *Enterobacteriaceae.* These relations were also identified by LEfSe analysis (Supplementary Fig. S3B). However, there were no differences in alpha diversity between groups (data not shown). Furthermore, we performed analysis of Spearman’s correlation coefficient in respect to body weight, blood glucose, gut permeability marker and gut microbiota members in the present cohort of animals (Supplementary Fig. S4A-C). Results indicated that the blood glucose level was positively associated with *Lactobacillus* (r = 0.787) and negatively with *Desulfovibrio* (r = − 0.89), while body weight was found to have negative correlation with such gut bacteria as *Enterobacteriaceae* and *Parabacteroides* (r = − 0.934 and r = − 0.802, respectively) (Supplementary Fig S4A,B). Uptake of FD4 to blood circulation, marker for the increased gut permeability was found positively correlated with abundance of mainly Gram-negative members, including *Proteobacteria* phylum and *Parabacteroides* genus (Supplementary Fig. S4C).

## Discussion

Alongside progressive central pathology resulting in neurodegeneration and cognitive decline^3,4^, HD pathology is defined by weight loss^27–29^. It has been pointed out that presymptomatic mutation carriers and patients with Huntington's disease show upper GI tract problems^30^, bladder^31^ and bowel dysfunction^32^ and that the severity of dysfunction correlates with poor quality of life and depression. Similarly, van der Burg and colleagues found impaired gut motility, diarrhea, and malabsorption of food in R6/2 mice^9^. In line with this, we here show decreased colon length in R6/2 mice.

Various diseases have been linked to intestinal epithelial barrier dysfunction and increased gut permeability: inflammatory bowel disease (IBD), irritable bowel syndrome (IBS), type 1 and type 2 diabetes^33^, depression^34^ and PD^35^. Here we report that R6/2 mice show an increase in gut permeability, which might contribute to the HD pathology. Interestingly, previous reports have shown that R6/2 mice have an impaired blood–brain barrier due to alteration in TJs expression in the BBB, in particular expression of occludin^10^. In this study, we used the R6/2 mice with a CAG repeat size range between 242 and 257, which leads to a slower disease progression compared to R6/2 mice with 150 CAGs^18^. Therefore, the lack of any alterations in occludin and ZO-1 protein levels might be due to the representation of an earlier disease stage compared to previous studies that showed a significant reduction in R6/2 TJ proteins in the brain^10,11^. It is known that, similar to the BBB, the intestinal integrity depends on tight junction proteins expression^5^, such as occludin, claudin and zonula occludens^6^. In our data there were no significant changes in tight junction protein (ZO-1 and occludin) expression at 12 and 16 weeks of age in R6/2 mice in colon, although at 16 weeks of age R6/2 mice exhibit an increased in gut permeability and dysbiosis. Since it has been shown that dysbiosis could contribute to re-arrangement of TJs which would, ultimately, lead to transient TJ disassembly and increase in intestinal permeability^36,37^, the dysbiosis found in this study could possibly be linked to the increased gut permeability in R6/2 mice. Interestingly, dysbiosis has been shown to affect levels of zonula occludens toxin (ZOT)^38^ which, in turn, increase the intestinal permeability by disengagement of ZO-1 from the tight junctional complex^39^. Ultimately, this influx would lead to irremediable gut changes and tissue damage. Indeed, histology using H&E in combination with occludin staining indicate that colon mucosal epithelia is disrupted in R6/2 mice at late disease stage.

In human GI tract the majority of bacterial species are represented by *Bacteroidetes* and *Firmicutes* phyla, as well as *Proteobacteria*, *Actinobacteria*, *Synergistetes*, and *Fusobacteria*^40–42^. There is an increased body of evidence that the GI microbiota is altered in diseases affecting the brain; Parkison’s disease^43^, mood disorders^44^, and autism^45^, as well as in other disorders like IBS^46^. In IBS, for example, there is a relative abundance of *Enterobacteriaceae (*proinflammatory bacterial species), while all short chain fatty acids (SCFAs) producers, such as *Lactobacillus*, *Bifidobacterium, Clostridiales* were in lower proportions in IBS patients^47^*.*

Evaluation of Gram + and Gram − bacteria provided us with the first hint on dysbiosis in R6/2 mice, which is in line with a previous study demonstrating that R6/1 male mice have gut dysbiosis^12^. While further evaluation of 16S rRNA sequenced data suggested dysbiosis, showing a significant difference between the two microbiomes, and, furthermore indicated a higher relative abundance of *Bacteroidetes* (Gram −) and lower relative abundance of *Firmicutes* (Gram +) in R6/2 mice as compared to WT littermates. Similar to previous findings where dysbiosis was associated with altered gut permeability^46,47^, our data indicated that reduction of body weight and increase in gut permeability was associated with the increased levels of Gram – bacteria. We have previously shown that gut transit time is increased in R6/2 mice^9^. Gastric emptying has to our knowledge not been investigated in R6/2 mice. We acknowledge that it is possible that intestinal physiological differences might affect the here obtained results.

Taken together, our data indicate an increased intestinal permeability in R6/2 mouse model of HD, together with significant changes in microbiota.

Further studies are needed to uncover the particular role of microbiota in HD disease progression and to develop novel and therapeutic ways to intervene early.

## Supplementary information

Supplementary Information.

## References

[1] HDCRG (1993). A novel gene containing a trinucleotide repeat that is expanded and unstable on Huntington’s disease chromosomes. The Huntington's Disease Collaborative Research Group. Cell.

[2] Beighton P, Hayden MR (1981). Huntington's chorea. S. Afr. Med. J..

[3] Giralt A, Saavedra A, Alberch J, Perez-Navarro E (2012). Cognitive dysfunction in Huntington's disease: humans, mouse models and molecular mechanisms. J. Huntingtons Dis..

[4] Pla P, Orvoen S, Saudou F, David DJ, Humbert S (2014). Mood disorders in Huntington's disease: from behavior to cellular and molecular mechanisms. Front Behav Neurosci.

[5] Ivanov AI (2012). Structure and regulation of intestinal epithelial tight junctions: current concepts and unanswered questions. Adv. Exp. Med. Biol..

[6] Ulluwishewa D (2011). Regulation of tight junction permeability by intestinal bacteria and dietary components. J. Nutr..

[7] Perez-Pardo P (2017). The gut-brain axis in Parkinson's disease: Possibilities for food-based therapies. Eur. J. Pharmacol..

[8] Clairembault T (2015). Structural alterations of the intestinal epithelial barrier in Parkinson's disease. Acta Neuropathol. Commun..

[9] van der Burg JM (2011). Gastrointestinal dysfunction contributes to weight loss in Huntington's disease mice. Neurobiol. Dis..

[10] Di Pardo A (2017). Impairment of blood-brain barrier is an early event in R6/2 mouse model of Huntington Disease. Sci. Rep..

[11] Drouin-Ouellet J (2015). Cerebrovascular and blood-brain barrier impairments in Huntington's disease: potential implications for its pathophysiology. Ann. Neurol..

[12] Kong G (2020). Microbiome profiling reveals gut dysbiosis in a transgenic mouse model of Huntington's disease. Neurobiol. Dis..

[13] Hsiao EY (2013). Microbiota modulate behavioral and physiological abnormalities associated with neurodevelopmental disorders. Cell.

[14] Cryan JF, de Wit H (2019). The gut microbiome in psychopharmacology and psychiatry. Psychopharmacology.

[15] Cryan JF (2019). The microbiota-gut-brain axis. Physiol. Rev..

[16] Mangiarini L (1996). Exon 1 of the HD gene with an expanded CAG repeat is sufficient to cause a progressive neurological phenotype in transgenic mice. Cell.

[17] Soylu-Kucharz R (2017). Neurofilament light protein in CSF and blood is associated with neurodegeneration and disease severity in Huntington's disease R6/2 mice. Sci. Rep..

[18] Morton AJ (2009). Paradoxical delay in the onset of disease caused by super-long CAG repeat expansions in R6/2 mice. Neurobiol. Dis..

[19] Gupta J, Nebreda AR (2014). Analysis of Intestinal Permeability in Mice. Bio-protocol.

[20] Sjogren M (2017). Ghrelin rescues skeletal muscle catabolic profile in the R6/2 mouse model of Huntington's disease. Sci. Rep..

[21] Fischer AH, Jacobson KA, Rose J, Zeller R (2008). Hematoxylin and eosin staining of tissue and cell sections. CSH Protoc..

[22] Kozich JJ, Westcott SL, Baxter NT, Highlander SK, Schloss PD (2013). Development of a dual-index sequencing strategy and curation pipeline for analyzing amplicon sequence data on the MiSeq Illumina sequencing platform. Appl. Environ. Microbiol..

[23] Caporaso JG (2010). QIIME allows analysis of high-throughput community sequencing data. Nat. Methods.

[24] Schmieder R, Edwards R (2011). Quality control and preprocessing of metagenomic datasets. Bioinformatics.

[25] Segata N (2011). Metagenomic biomarker discovery and explanation. Genome Biol..

[26] Bjorkqvist M (2005). The R6/2 transgenic mouse model of Huntington's disease develops diabetes due to deficient beta-cell mass and exocytosis. Hum. Mol. Genet..

[27] Pratley RE, Salbe AD, Ravussin E, Caviness JN (2000). Higher sedentary energy expenditure in patients with Huntington's disease. Ann. Neurol..

[28] Aziz NA (2010). Systemic energy homeostasis in Huntington's disease patients. J. Neurol. Neurosurg. Psychiatry.

[29] van der Burg JMM (2017). Body weight is a robust predictor of clinical progression in Huntington disease. Ann. Neurol..

[30] Andrich JE, Wobben M, Klotz P, Goetze O, Saft C (2009). Upper gastrointestinal findings in Huntington's disease: patients suffer but do not complain. J. Neural Transm. (Vienna).

[31] Kolenc M, Moharic M, Kobal J, Podnar S (2014). Bladder dysfunction in presymptomatic gene carriers and patients with Huntington's disease. J Neurol.

[32] Kobal J, Matej K, Kozelj M, Podnar S (2018). Anorectal dysfunction in presymptomatic mutation carriers and patients with Huntington’s disease. J. Huntingtons Dis..

[33] Fukui H (2016). Increased intestinal permeability and decreased barrier function: does it really influence the risk of inflammation?. Inflamm. Intest. Dis..

[34] Ohlsson L (2019). Leaky gut biomarkers in depression and suicidal behavior. Acta Psychiatr. Scand..

[35] Forsyth CB (2011). Increased intestinal permeability correlates with sigmoid mucosa alpha-synuclein staining and endotoxin exposure markers in early Parkinson's disease. PLoS ONE.

[36] Cenac N (2002). Induction of intestinal inflammation in mouse by activation of proteinase-activated receptor-2. Am. J. Pathol..

[37] Sturgeon C, Fasano A (2016). Zonulin, a regulator of epithelial and endothelial barrier functions, and its involvement in chronic inflammatory diseases. Tissue Barriers.

[38] Fasano A (1991). Vibrio cholerae produces a second enterotoxin, which affects intestinal tight junctions. Proc. Natl. Acad. Sci. USA.

[39] El Asmar R (2002). Host-dependent zonulin secretion causes the impairment of the small intestine barrier function after bacterial exposure. Gastroenterology.

[40] Ley RE, Turnbaugh PJ, Klein S, Gordon JI (2006). Microbial ecology: human gut microbes associated with obesity. Nature.

[41] Moore WE, Holdeman LV (1974). Human fecal flora: the normal flora of 20 Japanese-Hawaiians. Appl. Microbiol..

[42] Valdes AM, Walter J, Segal E, Spector TD (2018). Role of the gut microbiota in nutrition and health. BMJ.

[43] Scheperjans F (2015). Gut microbiota are related to Parkinson's disease and clinical phenotype. Mov. Disord..

[44] Callaghan BL (2020). Mind and gut: associations between mood and gastrointestinal distress in children exposed to adversity. Dev. Psychopathol..

[45] Sharon G (2019). Human gut microbiota from autism spectrum disorder promote behavioral symptoms in mice. Cell.

[46] Integrative HMPRNC (2019). The integrative human microbiome project. Nature.

[47] Pozuelo M (2015). Reduction of butyrate- and methane-producing microorganisms in patients with Irritable Bowel Syndrome. Sci. Rep..

