# Supplementary material for: Increased intestinal permeability and gut dysbiosis in the R6/2 mouse model of Huntington’s disease
Source: Sci Rep. 2020 Oct 26;10:18270. doi: 10.1038/s41598-020-75229-9 (PMC7589489; doi:10.1038/s41598-020-75229-9)
Supplement: Supplementary file 1 — Supplementary Information. [file 41598_2020_75229_MOESM1_ESM.pdf]

Increased intestinal permeability and gut dysbiosis in the R6/2 mouse model of Huntington´s disease

Tiberiu Loredan Stan[1*], Rana Soylu-Kucharz[1*], Stephen Burleigh[2], Olena Prykhodko[2], Ling Cao[2], Naomi Franke[1], Marie Sjögren[1], Caroline Haikal[3], Frida Hållenius[2] and Maria Björkqvist[1]

1. Wallenberg Neuroscience Center, Brain Disease Biomarker Unit, Department of Experimental Medical Sciences, Lund University, Lund, Sweden
2. Department of Food Technology, Engineering and Nutrition Lund University, Sweden
3. Neural Plasticity and Repair Unit, Wallenberg Neuroscience Center, Department of Experimental Medical Science, BMC A10, 221 84, Lund, Sweden

* These authors contributed equally to this work.

Supplement Table S1. Relative abundance of OTUs assigned at the phylum level.

**Relative abundance, %**	**R6/2**	**WT**
Actinobacteria	0.02 ± 0.00	0.06 ± 0.03
Bacteroidetes	61.93 ± 2.32	54.37 ± 5.73
Cyanobacteria	0.19 ± 0.17	0.25 ± 0.23
Deferribacteres	0.14 ± 0.08	0.64 ± 0.34
Firmicutes	34.28 ± 3.26	43.09 ± 5.70
Proteobacteria	3.15 ± 1.65	1.21 ± 0.40
Saccharibacteria (TM7)	0.09 ± 0.03	0.13 ± 0.05
Tenericutes	0.03 ± 0.03	0.03 ± 0.02
Verrucomicrobia	0.17 ± 0.03	0.2 ± 0.03

Results presented as mean ± SEM, (N= 4 R6/2 and 6 WT)

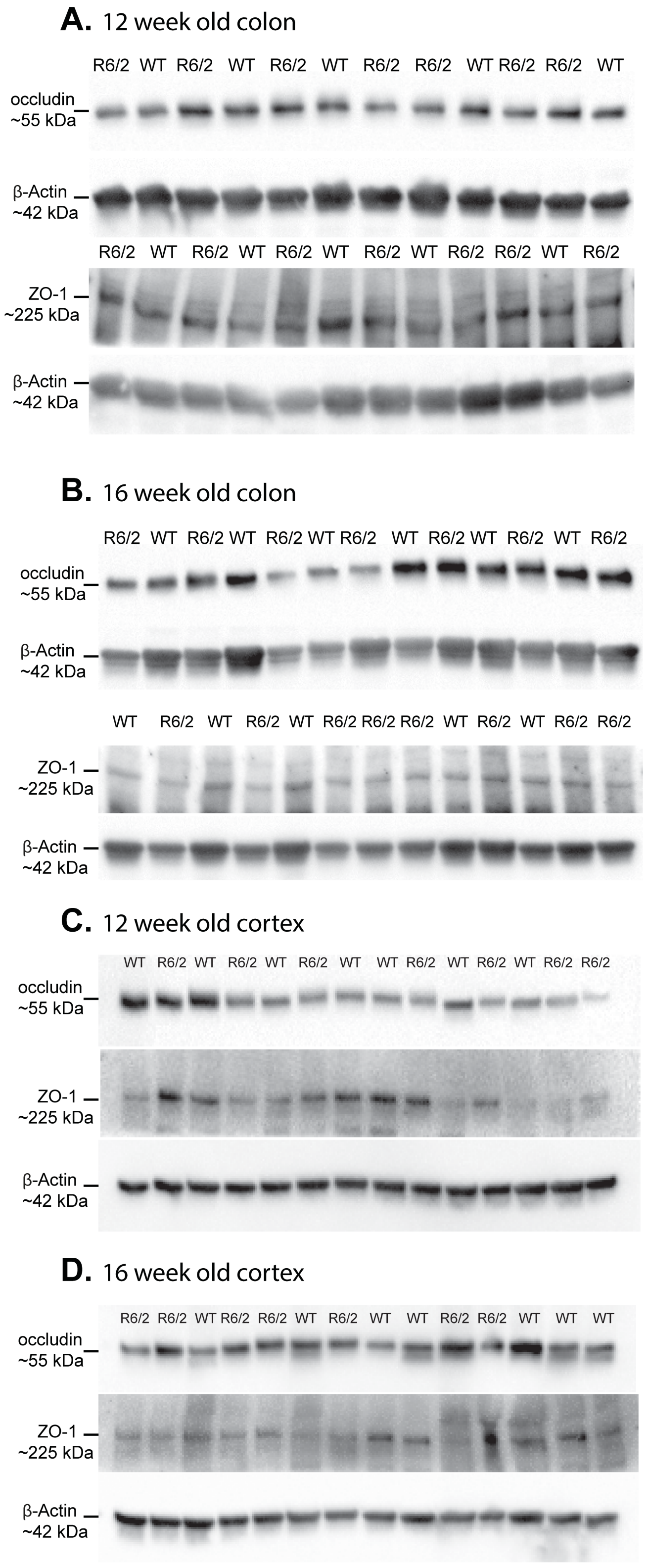

Supplementary Figure 1. Tight junction protein expressions in the colon and cortex. Western blot images showing the occludin, ZO-1 and β-actin protein expression in the colon at 12 **(A)** and 18 weeks **(B)** and in the cortex at 12 **(C)** and 16 **(D)** weeks of age.

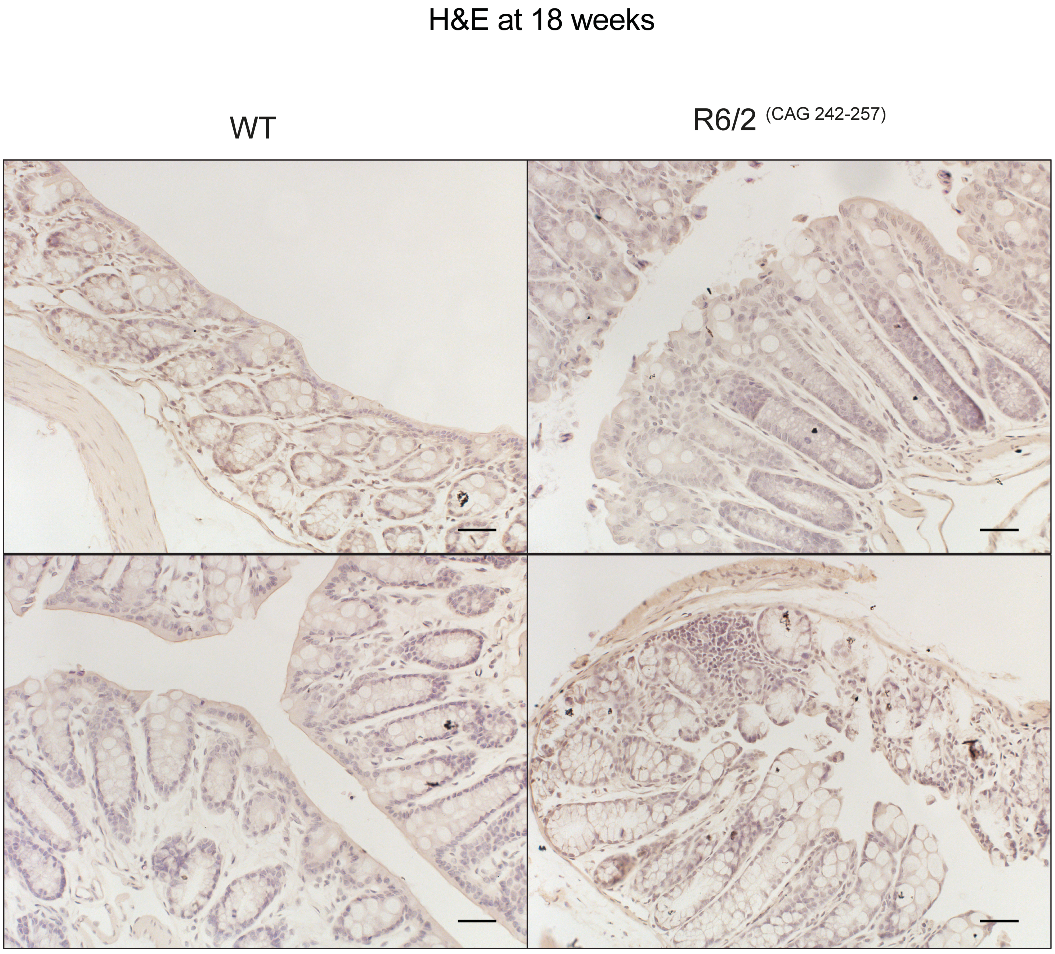

Supplementary Figure 2. H&E staining of colon from WT and R6/2 mice at 18 weeks of age (images were collected at 20x magnification). Scale bar represents 100 µm.

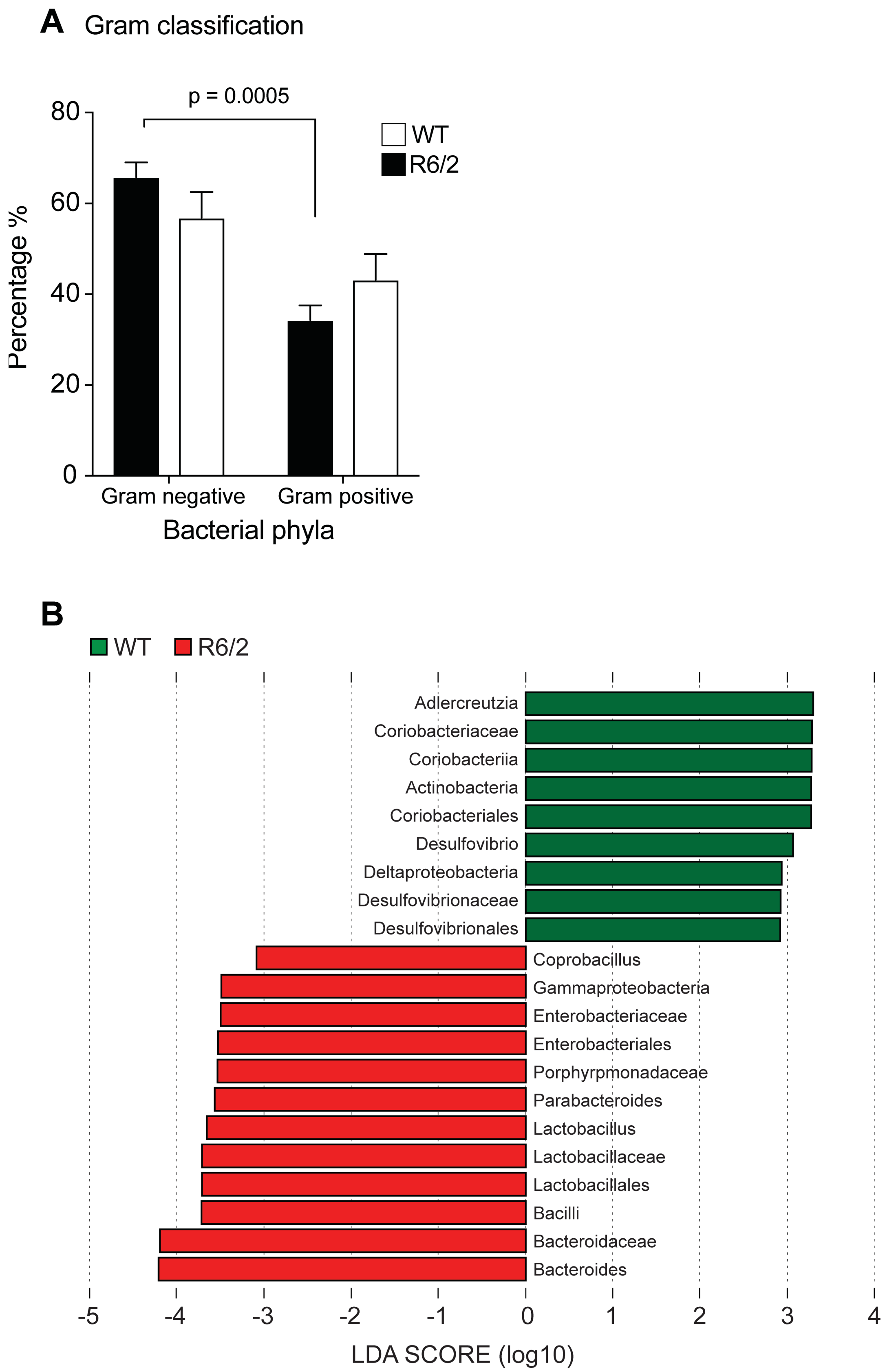

Supplementary Figure 3. Percentage of faecal bacteria after OTUs assignment to Gram-negative (Gram -) and Gram-positive (Gram +) phyla members in R6/2 (red) and

WT mice (green bars) **(A)**. LEfSe (Linear discriminant analysis effect size) was used to confirm *Bacteroides, Parabacteroides*, *Lactobacillus*, *Coprobacillus* and the *Enterobacteriaceae* as microbial biomarkers for the R6/2 genotype **(B)** (n= 4-6/genotype).

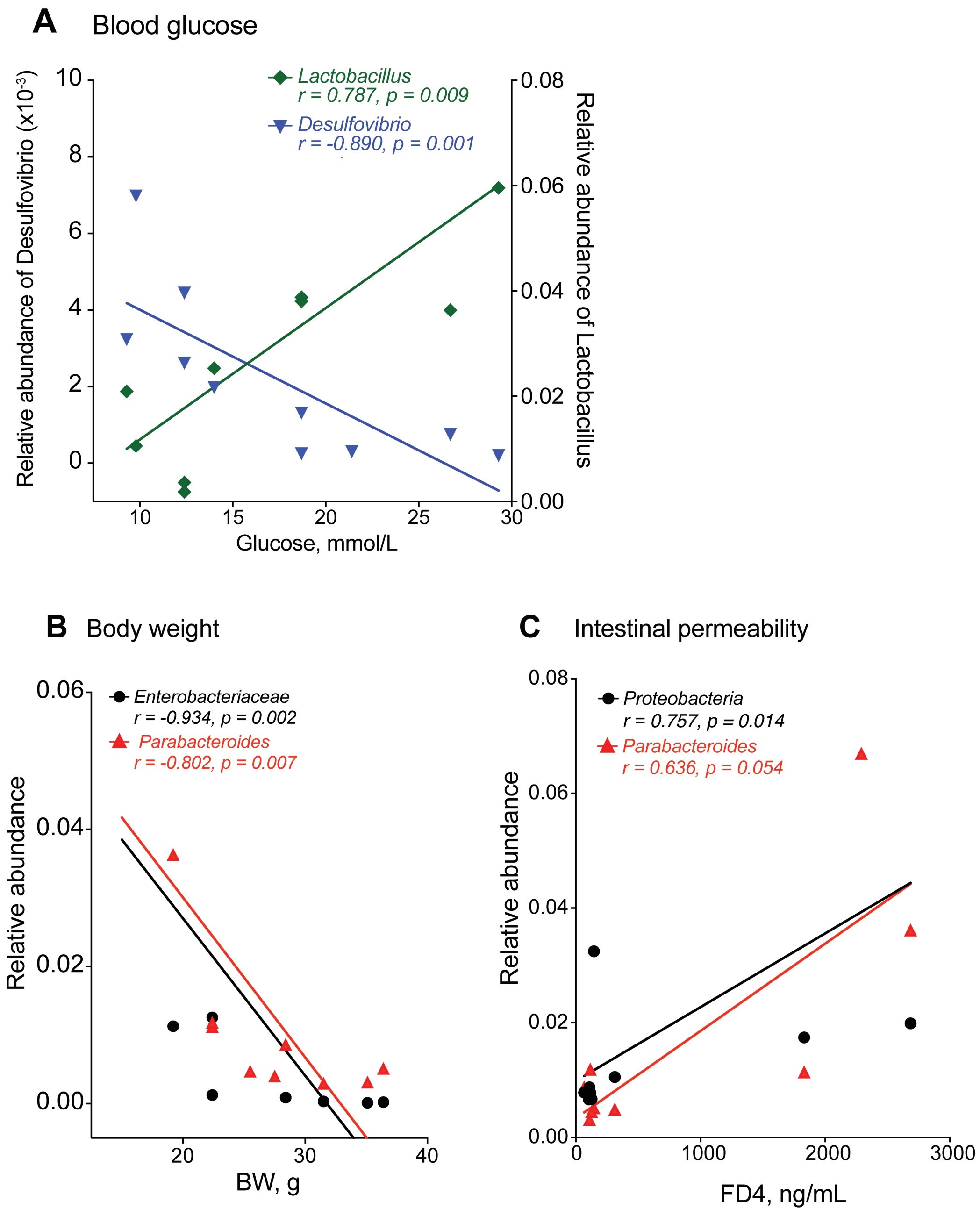

Supplementary Figure 4. Correlation analysis. Spearman's correlation between blood glucose **(A)**, body weight **(B)**, level of FD 4 passed to the blood circulation **(C)** and relative abundance of gut bacteria. (N= 4 R6/2 and 6 WT; 10 XY pair per test).
